# Unsupervised machine learning identifies clinically relevant patterns of CSF dynamic dysfunction in normal pressure hydrocephalus

**DOI:** 10.1016/j.clineuro.2025.109162

**Published:** 2025-09-15

**Authors:** Emanuele Camerucci, Petrice M. Cogswell, Jeffrey L. Gunter, Matthew L. Senjem, Matthew C. Murphy, Jonathan Graff-Radford, Ignacio Jusue-Torres, David T. Jones, Jeremy K. Cutsforth-Gregory, Benjamin D. Elder, Clifford R. Jack, John Huston, Hugo Botha

**Affiliations:** a Department of Neurology, Mayo Clinic, Rochester, MN, USA; b Department of Neurology, Kansas University Medical Center (KUMC), Kansas City, KS, USA; c Department of Radiology, Mayo Clinic, Rochester, MN, USA; d Department of Neurosurgery, Mayo Clinic, Rochester, MN, USA; e Department of Neurosurgery, Mayo Clinic Health System, Eau Claire, WI, USA

**Keywords:** INPH, DESH, Non-negative matrix factorization

## Abstract

**Background:**

Idiopathic normal pressure hydrocephalus (iNPH) is a common and debilitating condition whose diagnosis is made challenging due to the unspecific and common clinical presentation. The aim of our study was to determine if data driven patterns of cerebrospinal fluid (CSF) distribution can be used to predict iNPH diagnosis and response to treatment.

**Methods:**

We established a cohort of iNPH patients and age/sex-matched controls. We used Non-negative Matrix Factorization (NMF) on CSF probability maps from segmentation of T1-weighted MRI to obtain patterns or components of CSF distribution across participants and a load on each component in each participant. Visual assessment of morphologic phenotype was performed by a neuroradiologist, and clinical symptom improvement was assessed via retrospective chart review. We used the NMF component loads to predict diagnosis and clinical outcome after ventriculoperitoneal shunt placement for treatment of iNPH. Similar models were developed using manual Evan’s index and callosal angle measurements.

**Results:**

We included 98 iNPH patients and 98 controls split into test (20 %) and train (80 %) sets. The optimal NMF decomposition identified 7 patterns of CSF distribution in our cohort. Accuracy for predicting a clinical diagnosis of iNPH using the automated NMF model was 96 %/97 % in the train/test sets, which was similar to the performance of the manual measure models (92 %/97 %). Visualizing the voxels that contributed most to the NMF models revealed that the voxels most associated with a disproportionately enlarged subarachnoid space hydrocephalus (DESH) were the ones with higher probability of iNPH diagnosis. Neither NMF nor manual metrics performed well for prediction of qualitative clinical outcomes.

**Conclusions:**

NMF-generated patterns of CSF distribution showed high accuracy in discerning individuals with iNPH from controls. The patterns most relying on DESH features showed highest potential for independently predicting NPH diagnosis. The algorithm we proposed should not be perceived as a replacement for human expertise but rather as an additional tool to assist clinicians in achieving accurate diagnoses.

## 1. BACKGROUND

Normal pressure hydrocephalus (NPH) is a common condition with an estimated prevalence and incidence of 21.9 and 5.5 per 100,000, respectively [1]. It is clinically characterized by the insidious onset of the triad of cognitive impairment, gait impairment, and urinary incontinence. Diagnosis of NPH is challenging as these symptoms are exceedingly common in the older population [2,3] and overlap with those of neurodegenerative diseases. NPH is a potentially reversible form of cognitive impairment, if treated surgically via ventriculoperitoneal (VP) shunt [4]. As such, it is important to provide the necessary tools to perform a correct and timely diagnosis.

In the most recent international guidelines for NPH [4], this condition was divided into 4 main categories: idiopathic NPH (iNPH), secondary NPH (sNPH), congenital/developmental NPH, and familial NPH. The subtype that has recently received the greatest attention is iNPH, which has been additionally divided into 2 groups, based on the presence or absence of radiographic elements of disproportionately enlarged subarachnoid space hydrocephalus (DESH) [4]. The radiographic phenotype of DESH is defined by the presence of high-convexity tight sulci (HCTS), enlarged Sylvian fissures (ESF), and ventriculomegaly [5]. Being able to identify DESH may be particularly important, as shown in the SINPHONI-1 and 2 trials, where DESH iNPH patients showed higher responsiveness to VP shunt, as compared to non-DESH iNPH [6]. Another layer of complexity results from the fact that ventricular size and CSF volumes increase with age, and that DESH features can be present without overt symptoms.

Given these challenges [7,8], and the importance of making an accurate diagnosis of symptomatic iNPH given the therapeutic implications, it would be beneficial to provide physicians with automated tools that aid the detection of clinically manifested NPH. Since MR imaging is nearly always acquired in the evaluation of NPH, there is an opportunity to use advanced analytical techniques on MRI data to identify patterns that are predictive of iNPH and perhaps even response to treatment in iNPH.

Hence, the goals of this study are to use an unsupervised machine learning technique, non-negative matrix factorization (NMF), to identify low dimensional structure in the high dimensional MRI data and assess the ability of this to 1) differentiate patients with iNPH from controls and 2) predict clinical symptom improvement in iNPH patients after shunt placement.

## 2. Methods

### 2.1. Patients’ ascertainment

In our single center retrospective matched case-control study, patients were ascertained through the Clinical Data Linkage System at our institution using ICD-10 codes [9]. For inclusion, patients were required to have a diagnosis of iNPH and have been treated with ventriculoperitoneal shunt placement. For a diagnosis of iNPH we required a compatible clinical syndrome [4], a high-resolution MRI showing communicating hydrocephalus, normal opening pressure (<25 cm H2O), and an objective video improvement in gait after a high volume (>30cc) tap test. In addition, patients had to be above 50 years of age and have at least one MRI with a volumetric T1-weighted sequence (magnetization prepared rapid gradient echo, MPRAGE) available from prior to shunting. Exclusion criteria were secondary NPH (intracranial mass, prior intracranial intervention, history of meningitis, or intracranial hemorrhage) and congenital hydrocephalus (including longstanding overt ventriculomegaly of the adult, or LOVA). All diagnoses were performed via clinical impressions and neuroimaging was used as a tool to increase likelihood.

A cohort of age-matched cognitively unimpaired controls (CN) with a brain MRI, including the MPRAGE sequence, was identified from the Mayo Clinic Study of Aging, a population-based study of Olmsted County, MN [10]. We excluded controls with imaging features of DESH per dedicated image review (described below). DESH radiographic features are expected in a small portion of the control group based on prior work demonstrating asymptomatic DESH almost 7 % of the individuals aged 50 or older in the population-based Mayo Clinic Study of Aging cohort [11]. Importantly, these individuals are cognitively unimpaired based on consensus diagnosis, however, could have any other comorbidities. As the control cohort was derived from a community-based study, its inclusion provides a robust contrast to the tertiary-referral iNPH cases but also contributes to differences in baseline imaging and clinical profiles.

### 2.2. Standard protocol approvals, registrations, and patient consents

This study was approved by the Institutional Review Board of our institution.

### 2.3. Clinical symptoms abstraction

We abstracted the baseline presence and post-treatment change in the core clinical symptoms of NPH (cognitive impairment, urinary incontinence, gait difficulties) from the medical record. A physician (EC) accessed all patients’ charts and recorded presence/absence of any of the triad at time of shunt, as well as improvement (if any) at the 1-year follow up. All information gathered from this retrospective chart review was based on the neurologist’s history taking and exam, as well as patient’s and caregiver’s perception of improvement in gait, bladder, and cognitive function.

### 2.4. Image acquisition

The pre-shunt MRI closest to shunt date was used for phenotype analysis. If motion degraded, the second closest in time was obtained instead. All controls were scanned on 3.0 T GE (GE Healthcare, Waukesha, WI, USA) with the following parameters: TR/TE: ~7.0/~2.85 msec, TI: 900 msec, flip angle: 8^◦^, FOV: 256 × 256 mm^2^, slice thickness 1.2 mm, in-plane resolution 1.0 × 1.0, 166 slices. Owing to the clinical nature of the NPH scans, MRI scanners varied among patients, but the imaging protocols were similar (see Supplemental Tables 1–2 for details). Most NPH patients (N = 45) were scanned on 3.0 T Siemens (Munich, Germany) systems with comparable parameters (TI: 900–1000 msec, flip angle: 8–9^◦^, slice thickness 0.8–1.2 mm, in-plane resolution 0.9 ×0.9–1.2 × 1.2), followed by 1.5 T GE systems (N = 31) (TI: 900 msec, flip angle: 8–10^◦^, FOV: 256 × 256 mm^2^, slice thickness 1.2 mm, in-plane resolution 0.9 ×0.9156–166 slices), and 3.0 T GE systems (N = 21) (TI: 900 msec, flip angle: 8–10^◦^, FOV: 256 × 256 mm^2^, slice thickness 1–1.2 mm, in-plane resolution 0.9× 0.9–1 ×1156–190 slices). A handful of NPH patients were scanned on 1.5 T Siemens systems (N = 5) or 3.0 T Philips systems (N = 2).

### 2.5. Image processing

Details of our fully automated pipeline using Advanced Normalization Tools [12] and SPM12 [13] with in-house modifications and tools have been described previously [14]. Briefly, MPRAGE scans were normalized to the Mayo Clinic Adult Lifespan Template (MCALT; https://www.nitrc.org/projects/mcalt/) and segmented with MCALT priors/settings [15]. The spatial normalization parameters were then used to propagate the CSF probability maps to MCALT space, where they were smoothed at 4-mm full width at half-maximum (FWHM) prior to further MRI analyses.

### 2.6. Radiographic phenotype and measurements

A board certified neuroradiologist (PMC) with 5 years of experience performed visual review of all cases, blinded to clinical diagnosis or shunt response, to distinguish key radiographic features: Evan’s index (EI), callosal angle (CA), high convexity tight sulci (HCTS), enlarged sylvian fissures (ESF), and the presence of focally enlarged CSF spaces (focally enlarged sulci). These features were used to categorize patients into three radiographic phenotypes: DESH, defined as presence of HCTS and ESF; HCTS only, defined as a pattern without ESF; ventriculomegaly, defined as with Evan’s index > 0.3 without additional features. Of note, these radiographic phenotypes and measurements were made in the controls also, and controls were excluded if they were deemed to have radiographic features of DESH, but not if they had isolated ventriculomegaly, as it is a nonspecific finding.

### 2.7. Non-negative Matrix Factorization

Non-negative matrix factorization (NMF) is a type of unsupervised machine learning that identifies recurring spatial patterns in high-dimensional data (in this case, CSF voxel maps from MRI). This methodology is also useful for data reduction techniques and is specifically suited for purely positive data. Unlike supervised learning models, which require labeled outcomes, NMF only analyzes data structure and does not rely on pre-defined diagnostic categories.

Compared to other matrix decomposition methods like principal component analysis (PCA) or independent component analysis, NMF processes an input matrix structured by subject (row) against feature (column) and generates two novel matrices: a matrix structured by subject (row) against component (column) and another matrix shaped by component (row) to feature (column). The multiplication of these two matrices aims to be an approximation of the initial matrix. In neuroimaging contexts, features often represent voxel values corresponding to the relevant modality. The decomposition seeks to unearth a low-rank series of patterns in the voxel data that can largely account for variations across subjects. NMF’s unique trait compared to other methodologies is the positive-only constraint for matrices denoting subject-load and voxel-weight, meaning it enforces additive, parts-based representations, such that each basis function corresponds to a distinct pattern in data that contributes additively to reconstructing the original data. This makes NMF particularly well suited when working with tissue probability maps, as in our case, since these are bounded by zero at the low end. It also enhances factor readability compared to PCA, for example, which finds directions of maximal variance in the data, leading to components that mix positive and negative contributions, which makes it harder to directly interpret spatial patterns.

Numerous NMF algorithms have been developed, each tailored to distinct data types or properties. Our selection was the ’Minimum-volume rank-deficient’ algorithm [16] integrated into MATLAB [17,18], with minor customizations for our purposes. Recent evidence suggests that this algorithm maintains stability and provides a unique solution for any given input matrix [16]. In addition, it works in rank-deficient cases, which is likely true for neuroimaging data given the high degree of smoothing and spatial covariance.

The only adjustable parameter is the rank, denoting the number of components. To determine the optimal rank, we split our dataset into distinct training and test sets. The training set (a subset of participants used to learn patterns) was used exclusively for model development. The test set (a separate subset of participants not included during training) was held out for independent evaluation of model performance. The rank was obtained through a 5-fold cross-validation in the training set, described in more detail below, operating under the hypothesis that an optimal rank should meet two conditions: [1] the reconstruction error should be low in the dataset used to find the patterns (i.e., the key sources of variation are accounted for) and [2] the reconstruction error in an out of sample data set should not be inflated (i.e., we have not over-fit the discovery dataset by finding unique or noise components). In a scenario where the rank is under- or over-estimated then [1] will not be satisfied, whereas [2] will not be satisfied if over-fitting in the ascertainment dataset occurred.

With this in mind, we segmented the training set into five equal folds. The NMF algorithm was applied to four folds worth of data, and we then calculated the reconstructing error for these. We subsequently applied the fitted NMF model on the hold out fold to reconstruct its data. Reconstruction errors (absolute errors mean) were evaluated both for the discovery and the hold out fold. This cycle was executed five times, allocating each fold its turn as the isolated one. Given the randomness of fold divisions, the entire cross-validation process was also repeated five times. Consequently, each possible rank had 25 sets of discovery and hold-out reconstruction errors, all derived from the training set only. This exercise was performed for ranks between 3 and 10, after which we inspected reconstruction errors. Our aim was to find the rank that minimized the discovery reconstruction error but before the discovery and or hold-out reconstruction errors plateau or worsen (i.e., at an elbow). After identifying the optimal rank, we executed the NMF for the entire training dataset at the chosen rank. The voxel-weight matrix from training data was then applied to the test set via the identical NMF technique. The participant-component loads (both training and test) were used in subsequent statistical evaluations, and the voxel-weight matrix offered visual insights into the identified components. To reiterate, the 5-fold cross validation, performed a total of five times, and was all carried out using only the training data.

In order to contrast NMF not only with clinical measures but with other machine learning approaches, we calculated the computational DESH (cDESH) score as outlined in prior work [19]. This results in a single number for each participant, with values below 0 typically seen when DESH is absent and above 1 when DESH is present.

### 2.8. Statistical analysis

All statistical analyses were performed in R version 4.0.5. The two clinical groups (iNPH and control) were compared across demographic and imaging measures using Wilcoxon Rank Sum tests for continuous variables and Fisher’s exact test for binary variables. We also calculated the effect size and area under the ROC curve (AUC) for each individual imaging metric. The association between NMF components and radiographic phenotype and features was assessed using the Kruskal-Wallis test with post-hoc pairwise Wilcoxon tests. The level of significance was set at p < 0.05 for all tests. We used Holm’s method to correct p-values for multiple comparisons.

Pairwise comparisons were contingent on the Kruskal-Wallis for that measure being significant and were subsequently corrected for multiple comparisons using the Holm method. We also examined the association between component loads and demographic and radiographic features in the iNPH and control training sets separately (to avoid conflating a phenotype effect with a feature effect).

We then used the *glmnet* package to fit penalized regression (elastic net) models where the clinical phenotype or variable of interest was the outcome, and manual imaging quantified measures (Evan’s index, subcallosal angle) or component loads (all loads) were the predictors.

The manual imaging measure models were treated as the clinical reference standard in our analyses, as they represent expert-derived annotations used in current radiological workflows, whereas the NMF and cDESH models serve as automatic approaches that are scalable. The elastic net penalty, denoted by alpha in *glmnet*, controls the balance of L2 (ridge) and L1 (lasso) regularization. It was set to 0.2, i.e. favoring L2 (ridge) regularization over variable selection. The lambda parameter, which sets the strength of the regularization, was optimized using cross validation in the training set. For cDESH we used robust logistic regression as implemented in the *rlm* function in the *rms* package since elastic net requires multiple predictors. Lastly, we used elastic net models to predict individual binary manual measures of NPH as well as cDESH> 1 (i.e., positive) to examine whether NMF can capture these.

In all cases, separate models were fit in the training set for each clinical outcome of interest. These fitted models were then used to predict the clinical outcome in the test set as a true out of sample validation. To summarize the performance of each model we calculated the Matthew’s Correlation Coefficient (MCC) and balanced accuracy. Our focus was on the MCC given its desirable properties for binary classification tasks with imbalanced datasets. Specifically, the MCC ranges from −1 to 1, with 0 being the expected performance for a “coin tossing classifier” and higher values indicating models that can correctly predict most of the positive data instances. Accuracy was compared to the no-information rate as a null model, and we compared predictions from models using conventional measures with those using data driven components using McNemar’s test. For predicting shunt response, we also examined sensitivity, specificity, positive- and negative predictive values.

## 3. Results

### 3.1. Demographics and phenotype

We included 98 clinical iNPH patients in this study, 41 (42 %) of which were women. Overall, 79 were used as part of the train set, and 19 were used in the test set. They had a median age of 74 years. Cases were radiographically classified, according to phenotype, in: DESH (77 patients), HCTS (12 patients), ventriculomegaly (6 patients), none (3 patients). Detailed demographics divided by train/test sets are shown in Table 1. Importantly, we have individuals in our cohort that carry a clinical diagnosis of iNPH although with a phenotype described as “none”. This is related to the fact that such individuals were diagnosed *clinically* with iNPH and underwent shunt surgery, whereas the radiologist performing the above-mentioned classification was blinded on clinical history (iNPH vs control).

We included the same number of controls, of which 42 (43 %) were women. The median age was 74 years. Individuals were radiographically classified, according to phenotype, in: ventriculomegaly (19 patients) and none (79 patients). Detailed demographics divided by train/test sets are shown in Table 1.

### 3.2. NMF components

**Fig. 1.**
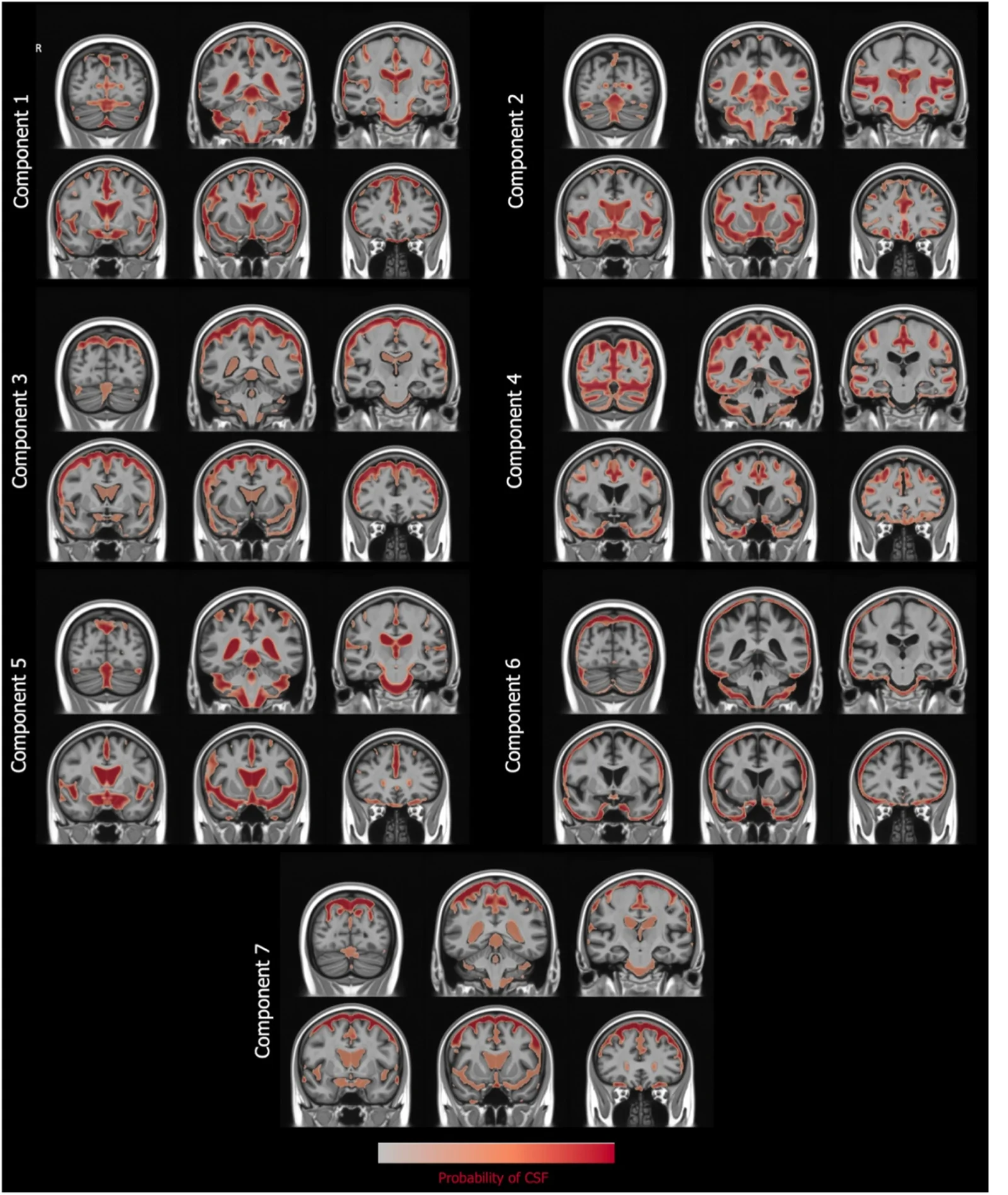
The seven NMF components, with darker shades of red indicating voxels with higher weight in each pattern.

**Fig. 2.**
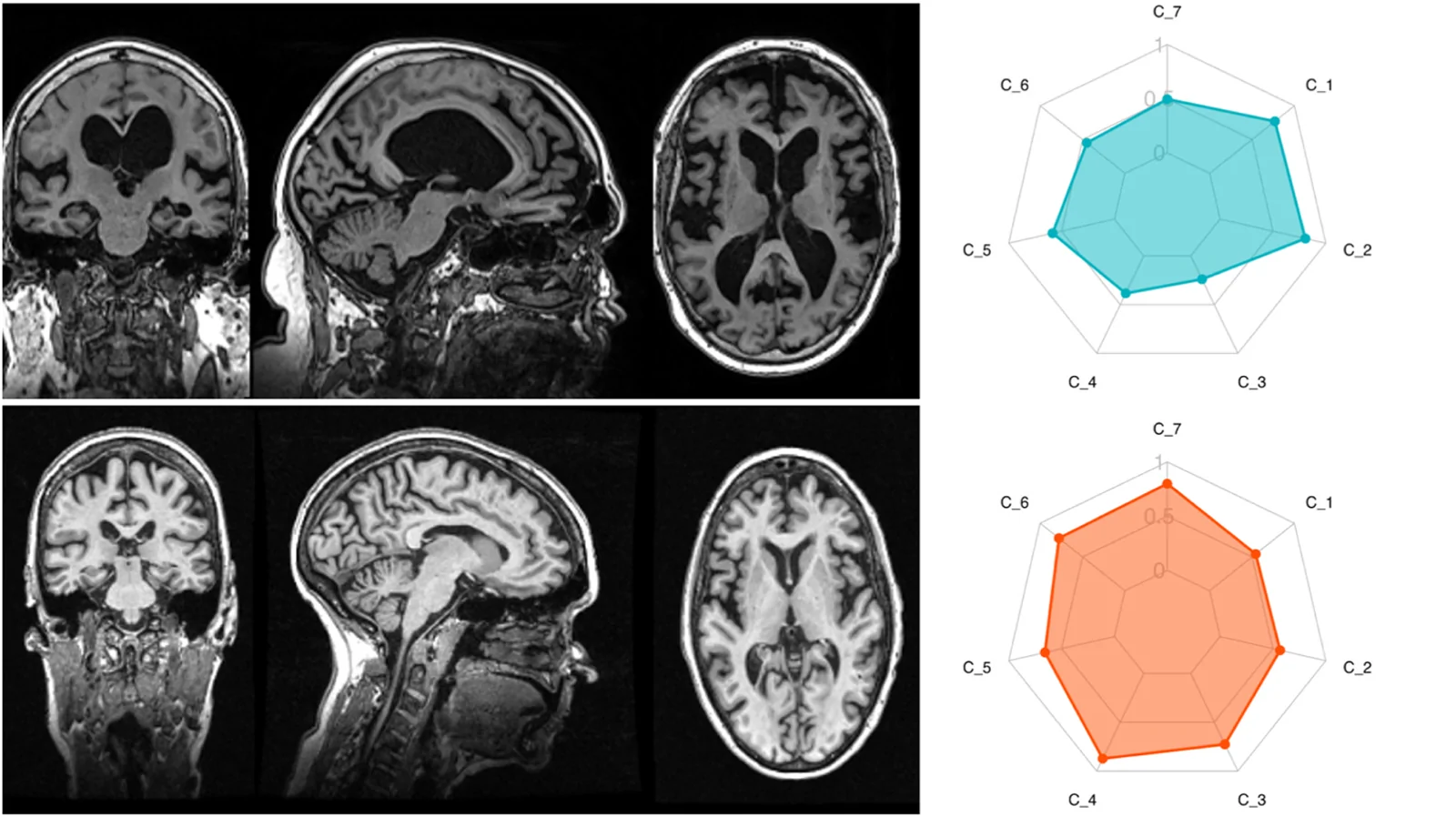
Example of 7 components’ loads in two individuals with opposite radiographic phenotype. The top case has iNPH with a DESH phenotype and loaded high on components 1 and 2 (high probability of CSF in the sylvian fissures and infra-sylvian sulci; low probability of CSF at the vertex) and low on 3, 4, and 6. The bottom case is a healthy control with no NPH features and loaded higher on 4, 6, and 7 and low on 1 and 2 (high probability CSF in the supra-sylvian sulci).

The optimal NMF decomposition was determined to be at a dimensionality of 7 components for CSF distribution (Supplemental Figure 1). The areas of maximal voxel weights for the 7 CSF distribution patterns are illustrated in Fig. 1. Visually, the dominant features of the components appear to be the following: component 1: ventricular, cisternal and sulcal CSF, including the Sylvian fissures; component 2: ventricular, cisternal, and sulcal CSF limited to the sylvian fissures, frontal and infra-sylvian sulci but with relative sparing of the high convexity sulci; component 3: ventricular, diffuse convexity, and juxta-dural CSF extending into the adjacent sulci; component 4: sulcal CSF with sparing of the sylvian fissures; component 5: ventricular, cisternal, and anterior sylvian fissures CSF; component 6: juxta-dural CSF, not extending into adjacent sulci; component 7: ventricular and high convexity sulci. Examples of individual participant loads on the 7 components together with representative views of their MRIs are shown in Fig. 2.

### 3.3. Association of NMF components with radiographic phenotype and features

Associations of the NMF components with demographic and manually assessed radiographic features are shown in Supplemental Table 3 for cases and Supplemental Table 4 for controls. In iNPH cases, we found: age was positively associated with loads on component 1 (adjusted p-value 0.02) and negatively associated with loads on component 2 (adjusted p-value 0.046); the presence of focally enlarged sulci was associated with lower loads on component 6 (adjusted p-value 0.04); enlarged Sylvian fissures was associated with lower loads on component 4 and 6; tight high convexity was associated with lower loads on component 6 (adjusted p-value <0.001).

Among controls: female sex was associated with lower loads on component 3 (adjusted p-value 0.02) and higher loads on component 4 (adjusted p-value 0.005); age was positively associated with loads on component 1, 3, and 6 (adjusted p-values <0.001, 0.02, <0.001); tight high convexity was associated with higher loads on components 1 (adjusted p-value 0.01) and 5 (adjusted p-value 0.03); other imaging associations were not significant.

**Fig. 3.**
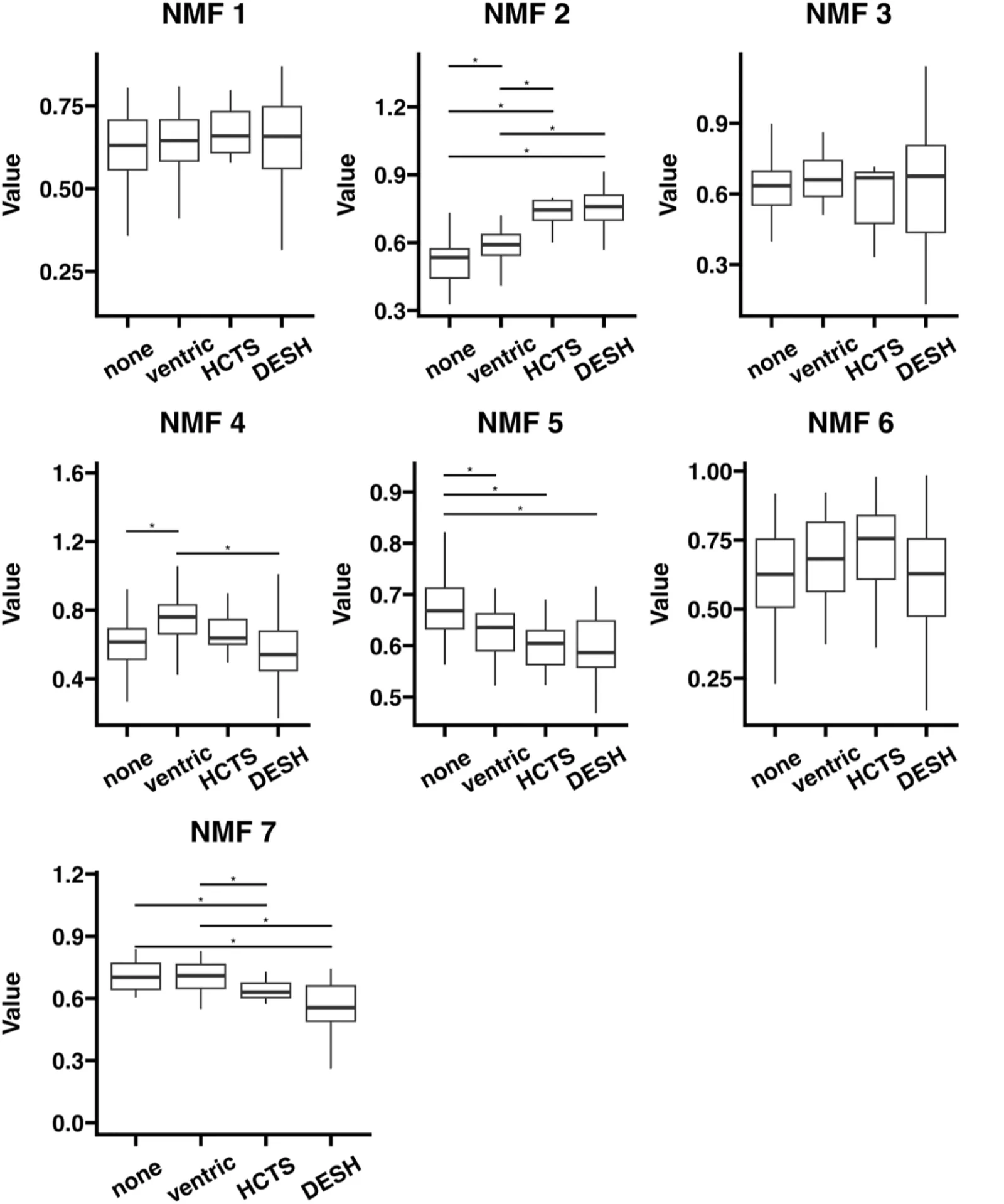
Association between NMF component loads and radiographic phenotype. An asterisk indicates a pairwise comparison that was significant (p < 0.05) after correction for multiple comparisons. *NMF= Non-Negative Matrix Factorization Component.

Fig. 3 represents the boxplot graphics of the association between the 7 automated NMF components and the radiographic phenotypes – ventriculomegaly, HCTS, DESH, and none – for iNPH patients and controls. Most notably, loads on component 2 – the one most strongly associated with a DESH pattern – significantly differed between isolated ventriculomegaly vs both HCTS and DESH. Similarly, loads on component 4 differed between isolated ventriculomegaly and both DESH and “none”. Finally, component 7 differed between DESH and HCTS from “none” and ventriculomegaly.

### 3.4. Single Measure Associations with Clinical NPH Diagnosis

Manual radiographic measures of iNPH, the cDESH score, and the 7 component loads are compared between patients with iNPH and controls in the training set in Table 2. The AUC for separating iNPH patients and controls using each measure as a single value is shown in the right-most column. Manual imaging measures and the cDESH score performed well, with an AUC above 0.91. Individual data driven components showed variable performance, ranging from excellent (0.96 for component 2) to close to 0.50 (components 1, 3, 4, and 6). We will focus here on the components that showed a difference between patients with iNPH and controls in Table 2.

### 3.5. Multivariate Models Predicting Clinical NPH Diagnosis

Results for models predicting iNPH vs CN using each manual measure, cDESH, and all NMF components are shown in Table 3. Performance for the NMF model was slightly higher than the manual model (0.96 vs 0.92). In the testing set, the manual model and NMF performed nearly perfectly; the cDESH model maintained a very high accuracy. This suggests all three approaches to predicting iNPH generalized well.

### 3.6. False positive and negative cases

**Fig. 4.**
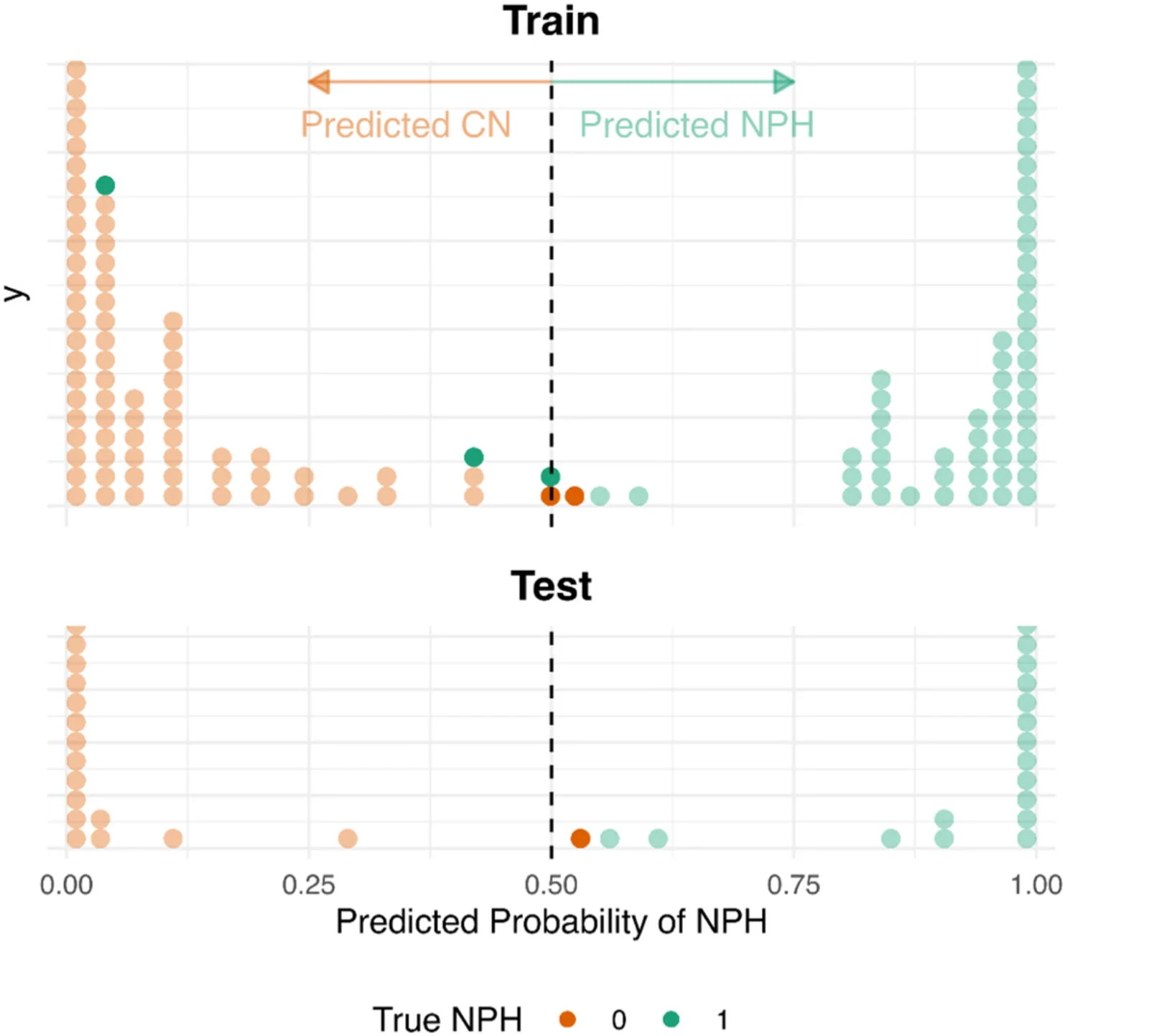
NMF based prediction of NPH in Train and Test sets. The X-axis represents the predicted probability of NPH. Whenever the probability was above 50 %, it was considered a “predicted NPH”. Vice versa, when it was below 50 %, that patient was considered a “predicted control”. Lightly colored green and orange dots represent, respectively, correctly allocated NPH and controls. Darker green/orange dots, on the other hand, represent NPH/control allocated in the wrong group.

Fig. 4 provides a graphical overview of the predicted probability of iNPH based on the NMF model in both train and test sets. There were 7 individuals that were incorrectly classified by the algorithm – 6/156 training patients and 1/38 test patients. Details regarding their radiographic phenotype, as well as predicted vs real diagnosis and probability of such are reported in Table 4. In brief, 4 of the iNPH cases were predicted as controls, all with non-DESH ventriculomegaly, whereas the remaining 3 were controls misclassified as NPH. Importantly, all the controls misclassified as iNPH had a probability near the decision boundary, perhaps related to a mild degree of some iNPH features – ventriculomegaly in one and an acute callosal angle in another.

### 3.7. Predicting symptom improvement and manual NPH markers

Among our 98 iNPH patients, there were 13 patients with no post-shunt clinical data, limiting symptom-based analysis to 85 patients (68 train, 17 test). At presentation, 65/68 (96 %) training cases had cognitive symptoms, 68/68 (100 %) had gait problems, and 50/68 (74 %) had urinary incontinence. One year after shunt placement, 27/65 (42 %) had cognitive improvement, 32/65 (49 %) had no improvement, and in 4 cases there was no data available regarding cognitive symptoms at follow up. In terms of gait difficulties, 46/68 (68 %) training cases had improvement in their clinical symptoms, while 22/68 (32 %) did not improve. Lastly, among those who had urinary symptoms in the pre-shunt period, 23/50 (46 %) improved at 1-year follow up, 23/50 (46 %) did not improve, and data on urinary improvement were not available in the remaining individuals.

When considering test cases, 13/17 (76 %) had cognitive symptoms at time zero; at the same visit, all test patients had gait impairment and 12/17 (71 %) had urinary symptoms. At 1-year follow up post shunt placement, improvement was documented for cognitive symptoms in 5/13 (38 %) cases, for gait in 13/17 (76 %), and for urinary symptoms in 5/12 (42 %) cases. The small test numbers precluded analyses of responses among the test cohort.

Table 5 shows the performance of manual, cDESH, and NMF based models in predicting clinical improvement in these 3 clinical symptoms. We also assessed the ability of the models to discern those with improvement in *any* domain vs those who are documented to have no improvement. The accuracy of cDESH measures to predict clinical improvement was at chance for all three symptoms on the training set. This is not surprising as this measure was explicitly designed to predict features of DESH in a binary manner rather than any response variables. Manual measures were also unable to predict any of the clinical symptoms. On the other hand, the NMF based model was able to predict urinary improvement with an accuracy of 0.63.

### 3.8. Summary of CSF regions contributing to NMF predictions

**Fig. 5.**
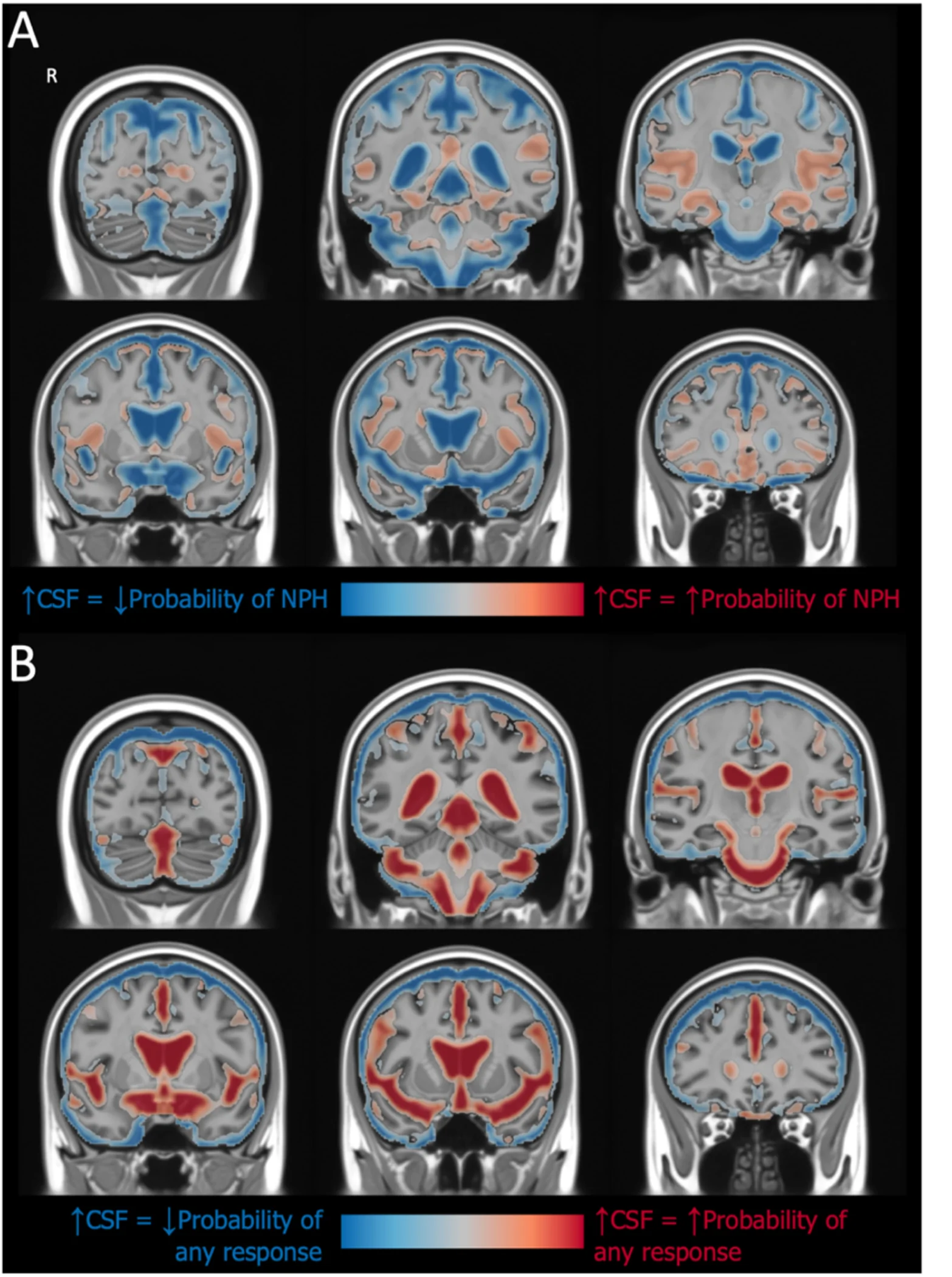
Voxel-level model coefficients for NMF based models. A: Coefficients for the model predicting clinical NPH diagnosis. Blue voxels indicate that a lower probability of CSF being present at the location is associated with a higher probability of clinical NPH, and red values indicate that higher probability of CSF is associated with a higher probability of clinical NPH. B: Coefficients for the model predict any clinical improvement at 1-year post-shunt. All coefficients were positive, with red values indicating that higher probability of CSF at that location is associated with a higher probability of improvement.

As we used linear models with a coefficient for each of the MRI components included in the model it is possible to propagate the estimated slope for each component back to voxel space to gain an understanding of which intracranial regions contributed the most to the model prediction for the presence of iNPH. We did so by multiplying the voxel-level weight maps for each component by the respective slope and then adding the resulting images together to get a single image (Fig. 5A). In this image, lower values (blue) indicate that CSF being present at the location is associated with a lesser probability of clinical iNPH being present, and higher values (red) indicate that CSF is associated with a greater probability of clinical NPH being present. Overall, the pattern reflecting DESH - increased CSF in the Sylvian fissures and major infra-Sylvian sulci, with crowding and hence reduced CSF at the vertex – was most strongly associated with a clinical diagnosis of iNPH. It was unexpected that some voxels in parietal and occipital sulci that often show focally enlarged sulci in DESH as well as the ventricular voxels were associated with a lower probability of iNPH. We suspect this may be driven by the model predicting *clinical* iNPH, meaning asymptomatic ventriculomegaly and cortical atrophy need to be discerned as causes of high CSF probability in these areas without iNPH necessarily being present. Fig. 5B shows the voxel-wise coefficient maps for the model predicting any improvement in any of the three clinical symptoms of NPH, which weighed CSF in the ventricles, Sylvian fissures, and basal cisterns heavily.

## 4. Discussion

In this study, we applied a NMF algorithm (a machine learning method for decomposing high-dimensional data into interpretable spatial patterns) to identify patterns of CSF distribution in iNPH. These patterns were then used to classify imaging data from patients with a clinical diagnosis of iNPH versus controls, and to predict clinical improvement after shunt placement. The primary findings were an accuracy of correctly classifying iNPH and controls at or above 96 % in both training and test sets, with high reliance on CSF voxels fitting with a radiographic pattern of DESH; and an accuracy of 63 % in predicting urinary symptoms improvement at 1 year. Our automated model performance compared well to manual features labeled by an expert radiologist and outperformed an existing automated marker for predicting response to shunting.

Prior research [20] has reported that combining two manual measures for diagnosing NPH (Evans index + callosal angle) may have an accuracy ranging between 89.6 % and 93.4 % in distinguishing patients with NPH from those without, consistent with our findings. In terms of prior automated approaches, one study used automated brain segmentation, and exhibited an accuracy of 96.3 % in distinguishing NPH from both AD patients and control subjects [21]. In contrast to our approach, this study used a white/gray matter segmentation map and included an AD cohort, a notable strength of the prior study. The accuracy using our automated model was of comparable strength in a much larger cohort, but we were also able to include a test set, which was of comparable size to the two studies [20,21].

Our group has previously evaluated a computational DESH score (cDESH) for detection of DESH in a community sample [19]. This model was based primarily on tight high convexity (due to the significant presence of the other DESH features in neurodegenerative conditions), and it showed that the most discriminant single regions were the left and right posterior callosomarginal fissures, with an overall AUC of detecting DESH of 0.99.

The strength of automated approaches, like NMF, does not rely only on the fact that it performed at least as well as the manual measures, but also that it requires less expertise (trained physician versus automated algorithm) and potentially less hands-on time and, as with other automated analyses, should decrease variability compared to having experts manually tracing conventional measures. Furthermore, the individual patterns offer a way to capture heterogeneity in iNPH and may show potential when predicting other quantitative measures of impairment or improvement in future work. Finally, we showed that the NMF models could predict most of the manually annotated measures with high accuracy in the test set, suggesting that it captures a lot of the same information (Supplemental Tables 3 and 4).

With regards of the seven components identified by the NMF algorithm, we found that most of them reflected a DESH pattern (represented by components 1, 2, 7 in Fig. 1), in line with the fact that the CSF voxels most associated with iNPH diagnosis were those typical of DESH (Fig. 4) – high probability of CSF in the sylvian fissures and infra-sylvian sulci and low probability of CSF at the vertex. This can be explained by the high prevalence of DESH radiographic features in our cohort (79 %), higher than what reported in the latest guidelines of the Japanese Society of NPH (64 %) for idiopathic cases [4]. A relatively high prevalence of DESH in our cohort of iNPH patients is driven by our practice’s focus on these cases as likely shunt responders as well as the fact that our institution is a tertiary referral center. Because of this high proportion of DESH features, our cohort is not powered to study NPH subtypes other than DESH.

The fact that our algorithm trained primarily on DESH iNPH cases likely explains some of the misclassified cases. We had 4 cases of iNPH erroneously classified as controls. All of them had non-DESH radiographic NPH phenotypes. On the other hand, the 3 controls misclassified as NPH had a NPH probability of approximately 50 %.

While we were able to separate controls from iNPH cases with high accuracy, none of the models here presented (manual, cDESH and NMF) were able to accurately predict clinical outcomes post-shunt, with the sole exception of our NMF model in predicting urinary symptoms improvement. Manual measures have previously demonstrated their unreliability as biomarkers for post-shunt clinical improvement [22,23]. Our NMF model had similarly poor accuracy for predicting cognitive and gait improvement, at 1-year follow up (respectively, accuracy of 51 % and 54 %), though it outperformed an existing automated marker of NPH (cDESH). Although prediction of overall clinical improvement remains limited, the model showed some potential in predicting urinary symptom improvement at the one-year post-shunt follow-up, achieving an accuracy of 63 %. We observed that higher presence of CSF within the infra-sylvian subarachnoid space, ventricular systems, and sylvian fissures were the areas with higher association with urinary improvement. When comparing to the available literature, a prior study [24] using an MRI-based automated algorithm (based on cortical volume, instead of CSF distribution) had better results in terms of predicting clinical response to shunt (gait AUROC= 0.88, urinary AUROC= 0.79, cognitive AUROC= 0.72).

It is worth noting that the manual and NMF models showed some potential when predicting a positive response but generally suffered when trying to identify non-responders. For instance, the positive predictive value (0.90) and specificity (0.80) of the NMF model for ‘any response’ was quite high whereas the negative predictive (0.36) and sensitivity (0.57) values were poor. This corresponds to 28/32 of the predicted responders being true responders, whereas only 13/36 predicted non-responders were true non-responders, and only 28/51 of true responders were identified by the model. Nevertheless, it does show the potential for identifying a subset of likely responders.

We acknowledge several limitations in our study: 1) We are aware of a dropout rate among individuals exhibiting a favorable response to shunt surgery, especially using a 1-year only follow up, potentially skewing our results toward individuals with an overall more severe clinical presentation. 2) We also acknowledge the inherent subjectivity in categorizing individuals as improving or non-improving in their clinical symptoms based on chart review only; however, a growing body of literature has shown that subjective improvement could predict stronger shunt response than objective measures [25]. A more objective methodology to assess clinical improvement can be considered for future studies. 3) Our cohort showed a high prevalence of DESH radiographic pattern due to the fact that our cohort of iNPH is comprised of individuals who underwent shunt surgery, and the DESH radiographic pattern has been shown to respond better to shunting [6]. 4) We did not include additional disorders mimicking iNPH. 5) We recognize our relatively short length of follow up (1 year only), mostly related to the fact that our patients were chosen from a recent cohort and hence did not have a lengthy history of NPH yet. 6) Lastly, we acknowledge that, as with all retrospective studies, the current results should be interpreted with caution until validated by prospective clinical trials designed to evaluate diagnostic sensitivity and specificity in a real-world setting.

In summary, we have presented an automated model to support the radiological assessment of iNPH, based on CSF distribution, featuring seven distinct CSF distribution patterns with an overall accuracy of 96 % in the training set and 97 % in the test set. Our model was primarily trained on cases exhibiting a DESH phenotype, which likely influenced its strong performance in classifying such cases, but limited its ability to generalize to non-DESH phenotypes. Despite its high radiological classification accuracy for iNPH, the algorithm displayed suboptimal capability in predicting future clinical improvement, except for urinary symptom improvement, where it achieved an accuracy of 63 %. We emphasize that this algorithm is not a replacement for clinical judgment or comprehensive diagnostic evaluation but may assist radiologists in identifying patterns consistent with DESH more objectively and reproducibly. Future work on automated detection of NPH should include cohorts of clinical and radiographic mimics, such as typical and atypical parkinsonian disorders and ex vacuo dilatation from cortically based degenerative disease.

## Supplementary Material

1

## Figures and Tables

**Table 1:** Demographics broken down by train and test sets.

	Train			Test		

Variable	CN	NPH	p	CN	NPH	p
Age	73.5 (6.1)	73.7 (6.4)	0.81	75.2 (8.8)	74.6 (4.3)	0.81
N Female	26 (33 %)	33 (42 %)	0.15	16 (84 %)	8 (42 %)	0.23
**Radiographic phenotype**						
HCTS	-	11	-	-	1	-
DESH	-	60	-	-	17	-
Ventriculomegaly	19	5	-	-	1	-
None	60	3	-	19	-	-

**CN** = cognitively normal, **NPH** = Normal pressure hydrocephalus, **DESH** = Disproportionately enlarged subarachnoid space hydrocephalus

**Table 2:** Comparison between continuous conventional measure of NPH and component loads between patients with NPH and controls in the training set including group median (IQR), the effect size for a comparison between means (Hedge’s g), and the AUC for separating patients and controls using each measure as a single value.

Variable	Control	NPH	P-value	Hedges	AUC

Evans index	0.27 (0.04)	0.36 (0.06)	< 0.001	−2.17 (−2.6; −1.8)	0.94 (0.9–0.94)
Callosal Angle	111.5 (18.2)	65.1 (27.8)	< 0.001	2.47 (2.1; 2.9)	0.95 (0.91–0.95)
cDESH Score	−2.369 (2.9)	4.34 (5.7)	< 0.001	-1.79 (−2.2; −1.4)	0.91 (0.86–0.91)
Component 1	0.63 (0.15)	0.66 (0.16)	0.39	−0.09 (−0.4; 0.2)	0.54 (0.45–0.54)
Component 2	0.55 (0.13)	0.74 (0.11)	< 0.001	−2.47 (−2.9; −2.1)	0.96 (0.94–0.96)
Component 3	0.63 (0.13)	0.68 (0.31)	0.25	−0.1 (−0.4; 0.2)	0.55 (0.46–0.55)
Component 4	0.64 (0.2)	0.61 (0.26)	0.24	0.08 (−0.2; 0.4)	0.55 (0.46–0.55)
Component 5	0.66 (0.07)	0.59 (0.09)	< 0.001	1.07 (0.7; 1.4)	0.78 (0.71–0.78)
Component 6	0.65 (0.24)	0.631 (0.34)	0.760	0.09 (−0.2; 0.4)	0.51 (0.42–0.51)
Component 7	0.71 (0.12)	0.585 (0.17)	< 0.001	1.29 (0.9; 1.6)	0.84 (0.78–0.84)

**NPH** = Normal pressure hydrocephalus, **AUC** = Area under the curve, **cDESH** = Computational disproportionately enlarged subarachnoid space hydrocephalus

**Table 3:** Overall performance for classifying iNPH vs control.

	Manual					cDESH			NMF				

Measure	N	N Present	Accuracy	MCC	P vs Null	Accuracy	MCC	P vs Null	Accuracy	MCC	P vs Null	P vs manual	P vs cDESH
Train	158	79	0.92	0.85	< 0.001	0.84	0.69	< 0.001	0.96	0.92	< 0.001	0.18	< 0.001
Test	38	19	0.97	0.95	< 0.001	0.90	0.79	< 0.001	0.97	0.95	< 0.001	1.00	0.25

**NPH** = Normal pressure hydrocephalus, **MCC**= Matthew’s Correlation Coefficient

**Table 4:** Overview of the seven cases misclassified by the NMF based model.

Case	Truth	Prediction	P (NPH)	Age	Sex	Phenotype	Evans index	Callosal angle

Train 1	NPH	Control	0.02	78.4	F	Ventriculomegaly	0.38	84.0
Train 2	NPH	Control	0.05	67.4	M	Ventriculomegaly	0.39	98.6
Train 3	NPH	Control	0.42	67.0	F	None	0.30	120.5
Train 4	NPH	Control	0.49	79.3	F	None	0.29	96.0
Train 5	Control	NPH	0.50	83.5	M	Ventriculomegaly	0.31	104.0
Train 6	Control	NPH	0.52	67.8	M	None	0.28	96.7
Test 1	Control	NPH	0.53	71.5	F	None	0.30	72.3

**NPH** = Normal pressure hydrocephalus, **DESH** = Disproportionately enlarged subarachnoid space hydrocephalus, **P (NPH)** = Probability of NPH

**Table 5:** Details of response prediction performance.

	Gait			Urinary			Cognitive			Any		

Measure	Manual	cDESH	NMF	Manual	cDESH	NMF	Manual	cDESH	NMF	Manual	cDESH	NMF
**N**	68			46			59			64		
**Prevalence**	0.68			0.50			0.46			0.77		
**Balanced Accuracy**	0.55	0.49	0.54	0.52	0.54	0.63	0.55	0.45	0.51	0.63	0.5	0.63
**P vs Null**	0.97	0.66	0.97	0.44	0.33	0.05	0.55	0.88	0.74	1.00	0.57	1.00
**MCC**	0.10	−0.01	0.08	0.04	0.09	0.26	0.09	−0.14	0.02	0.16	-	0.31
**Sensitivity**	0.61	0.98	0.63	0.57	0.52	0.70	0.63	0.11	0.56	0.65	1.00	0.57
**Specificity**	0.50	0.00	0.45	0.48	0.57	0.57	0.47	0.78	0.47	0.53	0.00	0.80
**PPV**	0.72	0.67	0.71	0.52	0.55	0.62	0.50	0.30	0.47	0.82	0.77	0.90
**NPV**	0.38	0.00	0.37	0.52	0.54	0.65	0.60	0.51	0.56	0.32		0.36

**NMF** = Non-negative matrix factorization, **cDESH** = Computational disproportionately enlarged subarachnoid space hydrocephalus, **PPV** = Positive predictive value, **NPV** = Negative predictive value, **MCC** = Matthew’s Correlation Coefficient

## Appendix A. Supporting information

Supplementary data associated with this article can be found in the online version at doi:10.1016/j.clineuro.2025.109162.
