# Supplementary material for: Unsupervised machine learning identifies clinically relevant patterns of CSF dynamic dysfunction in normal pressure hydrocephalus
Source: Clin Neurol Neurosurg. Author manuscript; Available in PMC 2026 Aug 20. (PMC13492978; doi:10.1016/j.clineuro.2025.109162)
Supplement: Supplementary file 1 [file NIHMS2182853-supplement-1.docx]

**Supplemental Table 1.** Details of MRI sequences in training set.

| **Vendor** | **Field Strength (T)** | **TE (ms)** | **TI (ms)** | **Flip angle (°)** | **Slice Thickness (mm)** | **Voxel Size (mm)** | **Slices** | **FOV (mm)** | **CN** | **NPH** |
| --- | --- | --- | --- | --- | --- | --- | --- | --- | --- | --- |
| GE | 1.5 | 4 | 1000 | 8 | 1.2 | 0.9 | 136 | 256 | 0 | 1 |
| GE | 1.5 | 4 | 1000 | 8 | 1.2 | 0.9 | 156 | 256 | 0 | 2 |
| GE | 1.5 | 4 | 1000 | 8 | 1.2 | 0.9 | 166 | 256 | 0 | 7 |
| GE | 1.5 | 4 | 1000 | 10 | 1.2 | 0.9 | 156 | 256 | 0 | 2 |
| GE | 1.5 | 4 | 1000 | 10 | 1.2 | 0.9 | 162 | 256 | 0 | 1 |
| GE | 1.5 | 4 | 1000 | 10 | 1.2 | 0.9 | 164 | 256 | 0 | 1 |
| GE | 1.5 | 4 | 1000 | 10 | 1.2 | 0.9 | 166 | 256 | 0 | 12 |
| GE | 3 | 2 | 900 | 8 | 1.2 | 0.9 | 156 | 256 | 0 | 2 |
| GE | 3 | 2 | 900 | 8 | 1.2 | 0.9 | 166 | 256 | 0 | 4 |
| GE | 3 | 2 | 900 | 8 | 1.2 | 0.9 | 196 | 256 | 0 | 2 |
| GE | 3 | 2 | 900 | 8 | 1.2 | 1 | 166 | 256 | 0 | 2 |
| GE | 3 | 3 | 900 | 8 | 1 | 1 | 190 | 256 | 0 | 2 |
| GE | 3 | 3 | 900 | 8 | 1.2 | 1 | 166 | 256 | 83 | 5 |
| Philips | 3 | 3 | - | 9 | 1 | 0.8 | 211 | 320 | 0 | 1 |
| Siemens | 1.5 | 2 | 1000 | 8 | 1 | 0.9 | 160 | 256 | 0 | 1 |
| Siemens | 1.5 | 3 | 900 | 8 | 1 | 1 | 176 | 256 | 0 | 1 |
| Siemens | 1.5 | 3 | 900 | 8 | 1 | 1 | 220 | 200 | 0 | 1 |
| Siemens | 1.5 | 4 | 1000 | 8 | 1 | 1.2 | 160 | 192 | 0 | 1 |
| Siemens | 3 | 2 | 1000 | 9 | 0.9 | 0.9 | 192 | 256 | 0 | 5 |
| Siemens | 3 | 2 | 1000 | 9 | 0.9 | 0.9 | 256 | 256 | 0 | 3 |
| Siemens | 3 | 2 | 1000 | 9 | 1.2 | 0.9 | 160 | 256 | 0 | 3 |
| Siemens | 3 | 3 | 900 | 9 | 0.9 | 0.9 | 216 | 256 | 0 | 3 |
| Siemens | 3 | 3 | 900 | 9 | 0.9 | 0.9 | 256 | 256 | 0 | 4 |
| Siemens | 3 | 3 | 900 | 9 | 0.9 | 1.2 | 256 | 193 | 0 | 1 |
| Siemens | 3 | 3 | 900 | 9 | 1.2 | 0.9 | 160 | 256 | 0 | 5 |
| Siemens | 3 | 3 | 945 | 9 | 0.8 | 0.8 | 240 | 300 | 0 | 9 |
| Siemens | 3 | 3 | 1000 | 9 | 0.9 | 0.9 | 192 | 256 | 0 | 1 |
| Siemens | 3 | 5 | 993 | 9 | 0.9 | 0.9 | 256 | 256 | 0 | 1 |

**Supplemental Table 2.** Details of MRI sequences in test set.

| **Vendor** | **Field Strength(T)** | **TE (ms)** | **TI (ms)** | **Flip angle (°)** | **Slice Thickness (mm)** | **Voxel Size (mm)** | **Slices** | **FOV (mm)** | **CN** | **NPH** |
| --- | --- | --- | --- | --- | --- | --- | --- | --- | --- | --- |
| GE | 1.5 | 4 | 1000 | 8 | 1.2 | 0.9 | 166 | 256 | 0 | 1 |
| GE | 1.5 | 4 | 1000 | 10 | 1.2 | 0.9 | 166 | 256 | 0 | 4 |
| GE | 3 | 2 | 900 | 8 | 1.2 | 0.9 | 166 | 256 | 0 | 1 |
| GE | 3 | 2 | 900 | 8 | 1.2 | 0.9 | 196 | 256 | 0 | 1 |
| GE | 3 | 2 | 900 | 8 | 1.2 | 1 | 166 | 256 | 0 | 1 |
| GE | 3 | 3 | 900 | 8 | 1.2 | 1 | 166 | 256 | 20 | 1 |
| Philips | 3 | 3 | - | 9 | 1 | 0.8 | 211 | 320 | 0 | 1 |
| Siemens | 3 | 2 | 900 | 8 | 1 | 0.9 | 160 | 256 | 0 | 1 |
| Siemens | 3 | 2 | 1000 | 9 | 0.9 | 0.9 | 256 | 256 | 0 | 1 |
| Siemens | 3 | 3 | 900 | 9 | 0.9 | 0.9 | 176 | 256 | 0 | 1 |
| Siemens | 3 | 3 | 900 | 9 | 0.9 | 0.9 | 216 | 256 | 0 | 1 |
| Siemens | 3 | 3 | 900 | 9 | 1.2 | 0.9 | 160 | 256 | 0 | 1 |
| Siemens | 3 | 3 | 945 | 9 | 0.8 | 0.8 | 240 | 300 | 0 | 4 |
| Siemens | 3 | 12 | - | 120 | 0.8 | 0.9 | 224 | 256 | 0 | 1 |

**Supplemental Table 3.**

Association between components and demographic and radiographic features among iNPH cases. Shown are median loads on each component, with an asterisk (*) indicating components where there was a statistically significant association (p-value <0.05, adjusted for multiple comparisons across components).

| **Variable** | Age | Sex | | **Focally enlarged sulci** | | **Cisterna magna** | | **Sylvians** | | **Aqueduct stenosis** | | **Tight convexity** | |
| --- | --- | --- | --- | --- | --- | --- | --- | --- | --- | --- | --- | --- | --- |
|  | r | Male | Female | No | Yes | No | Yes | No | Yes | No | Yes | No | Yes |
| **Component 1** | 0.33* | 0.7 | 0.65 | 0.65 | 0.69 | 0.67 | 0.76 | 0.64 | 0.68 | 0.68 | 0.61 | 0.56 | 0.69 |
| **Component 2** | -0.29* | 0.63 | 0.66 | 0.59 | 0.66 | 0.66 | 0.58 | 0.59 | 0.65 | 0.6 | 0.69 | 0.67 | 0.64 |
| **Component 3** | -0.02 | 0.66 | 0.51 | 0.56 | 0.63 | 0.57 | 0.69 | 0.66 | 0.59 | 0.59 | 0.66 | 0.44 | 0.61 |
| **Component 4** | -0.03 | 0.59 | 0.59 | 0.61 | 0.57 | 0.59 | 0.6 | 0.65 | 0.57* | 0.59 | 0.61 | 0.6 | 0.59 |
| **Component 5** | -0.03 | 0.65 | 0.63 | 0.62 | 0.65 | 0.63 | 0.65 | 0.63 | 0.64 | 0.63 | 0.66 | 0.63 | 0.63 |
| **Component 6** | 0.2 | 0.53 | 0.51 | 0.56 | 0.48* | 0.52 | 0.54 | 0.59 | 0.5* | 0.53 | 0.48 | 0.7 | 0.52* |
| **Component 7** | -0.02 | 0.6 | 0.6 | 0.59 | 0.61 | 0.6 | 0.61 | 0.58 | 0.61 | 0.59 | 0.61 | 0.57 | 0.61 |

**Supplemental Table 4.** Association between components and demographic and radiographic features among controls. Shown are median loads on each component, with an asterisk (*) indicating components where there was a statistically significant association (p-value <0.05, adjusted for multiple comparisons across components). Of note, radiographic NPH markers were rare in controls, resulting in asymmetric comparisons (e.g., for enlarged Sylvian’s, 80 controls lacked the findings and only 3 were positive for it).

| **Variable** | Age | Sex | | **Focally enlarged sulci** | | **Cisterna magna** | | **Sylvians** | | **Aqueduct stenosis** | | **Tight convexity** | |
| --- | --- | --- | --- | --- | --- | --- | --- | --- | --- | --- | --- | --- | --- |
|  | r | Male | Female | No | Yes | No | Yes | No | Yes | No | Yes | No | Yes |
| **Component 1** | 0.69* | 0.53 | 0.48 | 0.52 | 0.76 | 0.52 | 0.52 | 0.51 | 0.70 | 0.52 | 0.69 | 0.51 | 0.72* |
| **Component 2** | -0.05 | 0.61 | 0.65 | 0.63 | 0.59 | 0.61 | 0.66 | 0.63 | 0.56 | 0.63 | 0.47 | 0.63 | 0.59 |
| **Component 3** | 0.32* | 0.68 | 0.56* | 0.64 | 0.77 | 0.64 | 0.63 | 0.64 | 0.67 | 0.64 | 0.60 | 0.64 | 0.69 |
| **Component 4** | -0.13 | 0.64 | 0.73* | 0.66 | 0.62 | 0.66 | 0.68 | 0.66 | 0.36 | 0.66 | 0.32 | 0.66 | 0.46 |
| **Component 5** | 0.23 | 0.54 | 0.60 | 0.55 | 0.76 | 0.55 | 0.56 | 0.54 | 0.74 | 0.55 | 0.74 | 0.54 | 0.78* |
| **Component 6** | 0.51* | 0.67 | 0.70 | 0.67 | 0.58 | 0.67 | 0.67 | 0.67 | 0.54 | 0.67 | 0.32 | 0.67 | 0.54 |
| **Component 7** | -0.26 | 0.61 | 0.58 | 0.60 | 0.54 | 0.60 | 0.60 | 0.60 | 0.59 | 0.60 | 0.66 | 0.60 | 0.57 |

**Supplemental Figure 1.** Reconstruction error for the discovery and hold out folds on the training set for a range of NMF decomposition ranks. The dotted line indicates the chosen rank for our paper.
